# Effects of Fermentation Time and Type of Tea on the Content of Micronutrients in Kombucha Fermented Tea

**DOI:** 10.3390/nu14224828

**Published:** 2022-11-15

**Authors:** Karolina Jakubczyk, Patrycja Kupnicka, Klaudia Melkis, Oliwia Mielczarek, Joanna Walczyńska, Dariusz Chlubek, Katarzyna Janda-Milczarek

**Affiliations:** 1 Department of Human Nutrition and Metabolomics, Pomeranian Medical University in Szczecin, 24 Broniewskiego Street, 71-460 Szczecin, Poland; 2 Department of Biochemistry and Medical Chemistry, Pomeranian Medical University, 72 Powstańców Wielkopolskich Street, 70-111 Szczecin, Poland

**Keywords:** kombucha, fermentation, microelements

## Abstract

The fermented tea beverage Kombucha is obtained through a series of biochemical and enzymatic reactions carried out by symbiotic cultures of bacteria and yeasts (SCOBY). It contains organic acids, vitamins, amino acids, and biologically active compounds, notably polyphenols, derived mainly from tea. Kombucha exhibits a range of health-promoting properties, including antioxidant or detoxifying effects. This fermented beverage is traditionally brewed with black tea, but other types of tea are used increasingly, which may have significant implications in terms of chemical composition and health-promoting effects. In this preliminary study, we investigated the content of micronutrients (manganese (Mn), copper (Cu), iron (Fe), chromium (Cr) and zinc (Zn)) by the ICP-OES method in Kombucha prepared with black, red, green and white tea at different time points of fermentation (1, 7, 14 days). It should be noted that the composition of separate ingredients such as tea, leaven or sugar has not been studied. Kombucha had the highest content of zinc—0.36 mg/L to 2.08 mg/L, which accounts for between 3% and 26% of the RDA (Recommended Dietary Allowance) for adults, and the smallest amounts of chromium (0.03 mg/L to 0.09 mg/L), which however represents as much as between 75% and 232% of the RDA. It has been demonstrated that the type of tea as well as the day of fermentation have a significant effect on the concentrations of selected minerals. Kombucha can therefore supplement micronutrients in the human diet.

## 1. Introduction

Kombucha is a low-alcohol beverage made by fermenting a sugared tea infusion with symbiotic cultures of bacteria and yeasts (SCOBY), commonly called “tea fungus”. This complex community comprises acetic acid bacteria (AAB) (*Gluconobacter: G. entanii, G. oxydans, Acetobacter: A. xylionoides, A. aceti, A. pasteurianus*)*, Komagataeibacter (K. intermedius, K. rhaeticus*), lactic acid bacteria (LAB) (*Lactobacillus* and *Leuconostoc*)*,* and yeasts (*Schizosaccharomyces pombe, Zygosaccharomyces bailii, Saccharomyces*). Its dynamics are still not fully understood [1,2,3,4,5,6,7].

**Figure 1:**
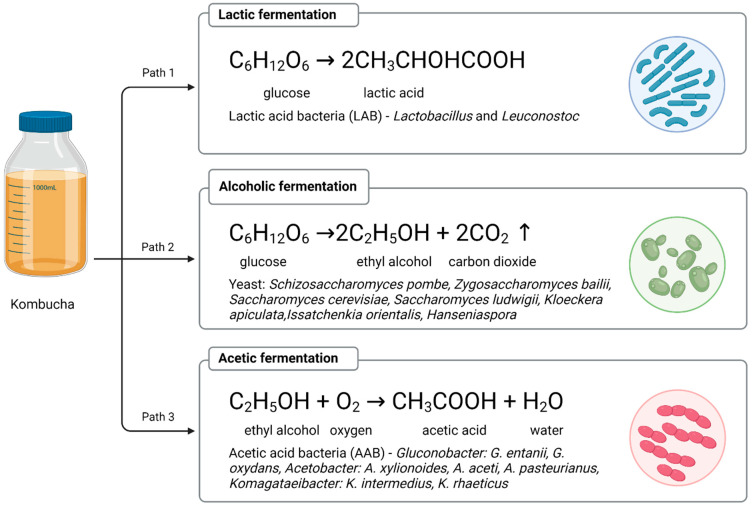
Types of fermentation in Kombucha. Created with BioRender.com.

Kombucha fermentation is a combination of three fermentation processes: alcoholic, lactic and acetic acid. The bacteria present in the tea fungus are responsible for the production of acetic acid, while yeasts, representatives of the osmophilic type, induce the breakdown of sucrose. The resulting product, glucose, is a substrate for both lactic fermentation and alcoholic fermentation [4,8]. Under the influence of lactic acid bacteria, glucose is converted into lactic acid. During alcoholic fermentation, on the other hand, glucose is converted into ethyl alcohol, releasing carbon dioxide. Ethanol produced during the breakdown of glucose is oxidised by acetic acid bacteria to acetic acid and acetaldehyde (Figure 1).

*Acetobacter* are also responsible for the oxidation of glucose to glucuronic and gluconic acid, the key detoxifying agents in Kombucha. The process is also accompanied by the production of cellulose, which is part of the tea fungus [7]. Thus, sweetened tea is transformed into Kombucha by a process involving three types of fermentation, whose activity and dominance changes over time. Initially, the beverage is rich in glucose, followed by alcohol, while the final stage is dominated by organic acids, including acetic acid.

The elementary ingredients of the traditional recipe are black tea and white sugar [9]. Sucrose is the main source of carbon in Kombucha fermentation due to its uncomplicated structure and ability to provide simple carbohydrates for microbial metabolic pathways, as well as its low cost and easy availability [1,10]. However, Kombucha is more and more often made with green, red and white teas or herbal infusions instead of black tea [7], and with coconut sugar, cane sugar, maple syrup or honey instead of white sugar [1,10,11]. The entire Kombucha production process takes place at room temperature over 7–14 days, during which it acquires its distinct chemical and organoleptic characteristics. The flavor of the finished tea beverage is described as mildly sour, fruity, fizzy, resembling that of cider [12,13,14].

Research to date indicates that the fermented tea beverage contains numerous bioactive substances, originating from the material used, mainly tea, but also resulting from the enzymatic transformations of organic compounds carried out by microorganisms. The bioactive compounds include vitamins (E, K, B, C), amino acids (especially theanine, a derivative of glutamine), polyphenolic compounds, i.e., catechins and flavonoids, and a variety of minerals [4,7,15,16,17].

It is worth noting that Kombucha has been hailed as a functional fermented beverage with antioxidant, antimicrobial, antioxidant, anti-diabetic properties, reducing cholesterol levels, supporting immune and digestive function, and also stimulating liver detoxification [7,17,18,19]. However, the presence and the amounts of nutrients in Kombucha, and accordingly its beneficial effects, are determined by a number of factors, such as the parameters of the fermentation process, e.g., time, or the ingredients used [12].

In addition, Kombucha is gaining popularity as one of the novelties offered by fermented food manufacturers. The valuable composition of such foods translates into manifold biological effects in consumers’ bodies [20]. As a result of the biochemical transformations of organic compounds by microorganisms, it is possible to obtain foods with not only extended shelf life and microbiological stability, but also a higher nutritional value. The fermentation process largely breaks down the antinutrients in food, e.g., phytates, enhancing the potential for utilizing nutrients of key importance in the human diet. Also, fermented foods may be better tolerated by people with certain food sensitivities and intolerances [21]. Recent scientific reports confirm the interactions between microorganisms and plant products [22,23]. The microbiota present in the product is responsible for “digesting” plant material into absorbable active small molecules, which then induce physiological changes in the body [24,25,26,27]. Research findings show that fermentation of tea residues can significantly increase the antioxidant activity (up to 3.25 times), as well as the polyphenol concentrations (5.68 times) of Kombucha. Interestingly, green tea residues showed a stronger effect than those of black tea [26].

Even though Kombucha is gaining increasing recognition and the range of flavors available on the food market continues to expand, the detailed composition and the effects of individual fermentation parameters or type of tea on the properties of this product are not fully understood [12]. A few scientific articles report that Kombucha is a source of micronutrients, but the data are incomplete. Hence, the aim of this study was to evaluate for the first time the content of selected micronutrients in Kombucha beverages made with black, green, white and red tea at different time points of fermentation.

## 2. Materials and Methods

### 2.1. Plant Material

The material consisted of four types of leaf tea (*Camellia sinensis*): black, green, white and red (Pu-ERH) originating in China.

### 2.2. Preparation of Kombucha

**Figure 2:**
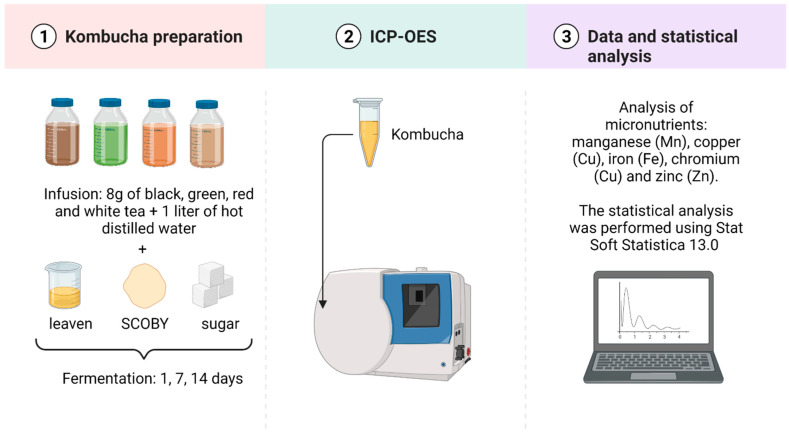
Material and methods—Preparation of Kombucha and laboratory analyses. Created with BioRender.com.

The Kombucha cultures in the present article were purchased from a commercial shop. Kombucha bacterium component belongs to the strains of *Acetobacter*, while the yeasts are *Saccharomyces cerevisiae* and *Zygosaccharomyces*. One hundred grams of sugar (100.0 g/L, 10.0%), eight grams of tea (8.0 g/L, 0.8%) and 1 L of hot distilled water (90 °C) were added to the flask. The solution was infused for 10 min in a sterile conical flask. After cooling to 30 °C, the tea decoction was filtered into clean glass bottles and Kombucha pellicle (100.0 g/L, 10.0%) and one hundred milliliters of leaven from a previous culture (100.0 mL/L, 1.0%) were added (Figure 2).

### 2.3. Fermentation of Kombucha

Kombucha culture was kept under aseptic conditions. Fermentation was carried out by incubating the Kombucha culture at 28 ± 1 °C for 1, 7 and 14 days. Replicates were prepared so that each replicate was completely collected after its stipulated period of fermentation. The Kombucha obtained was filtered and analyzed.

### 2.4. Determining Elements Content in Infusions

Sample preparation:

The samples were mineralized using the MARS 5 CEM microwave digestion system. The volume of the sample given to research was 0.8 mL. The samples were transferred to clean polypropylene tubes, 2 mL of 65% HNO_3_ (Suprapur, Merck, Darmstadt, Germany) was added to each vial and each sample was allowed 30 min pre reaction time in the clean hood. After completion of the pre-reaction time, 0.5 mL of non-stabilized 30% H_2_O_2_ solution (Suprapur, Merck, Darmstadt, Germany) was added to each vial. Once the addition of all reagents was complete, the samples were placed in special Teflon vessels and heated in the microwave digestion system for 35 min at 180 °C (15 min ramp to 180 °C and maintained at 180 °C for 20 min). At the end of digestion all samples were removed from the microwave and allowed to cool to room temperature. In the clean hood, samples were transferred to acid-washed 15 mL polypropylene sample tubes. A further 5-fold dilution was performed prior to ICP-OES measurement. A volume of 2 mL was taken from each digest. The samples were spiked with an internal standard to provide a final concentration of 0.5 mg/L Ytrium, 1 mL of 1% Triton (Triton X-100, Sigma, Kawasaki, Japan) and diluted to the final volume of 10 mL with 0.075% nitric acid (Suprapur, Merck, Darmstadt, Germany). Blank samples were prepared by adding concentrated nitric acid (500 µL) to tubes without a sample and subsequently diluted in the same manner described above. Multielement calibration standards (ICP multi-element standard solution IV, Merck, Darmstadt, Germany) were prepared with different concentrations of inorganic elements in the same manner as in blanks and samples. Deionized water (Direct Q UV, Millipore, Burlington, MA, USA, approximately 18.0 MΩ) was used for preparation of all solutions.

Sample determination:

Samples were analyzed using inductively coupled plasma optical emission spectrometry (ICP-OES, ICAP 7400 Duo, Thermo Scientific), which is often utilized to measure the concentrations of mineral nutrients as well as heavy metals and allows simultaneous measurements of many different elements, also in plant samples [28,29]. ICP-OES with a concentric nebulizer and cyclonic spray chamber was used to determine the content of micro and macroelements. The analysis was performed in both radial and axial modes. The wavelengths used in the analysis were: Zn 206.200, Cr 205.560, Mn 257.610, Cu 224.700, Fe 259.940.

Validation was performed by evaluating the following: NIST SRM 8414 reference material (National Institute of Standards and Technology, USA), limit of detection (LOD), and the recovery of internal standard (yttrium). To eliminate possible interference, the emission lines were selected empirically in pilot measurements. This model of validation is often used in ICP-OES studies, also those regarding plant samples [29]. The recovery of Y was within 90–106%. The R2 values for all standard curves were in the range between 0.998 and 1.000.

### 2.5. Statistical Analysis

All determinations were carried out in at least three replicates. Statistical analysis was performed using Stat Soft Statistica 13.0 and Microsoft Excel 2017. Distributions of values for individual parameters were analyzed using the Shapiro-Wilk test. Since the distribution of continuous variables deviated from normal, the Kruskal-Wallis test was used to evaluate the differences between the studied parameters. Spearman’s correlation test was used to determine the correlations between the parameters studied. Results were expressed as mean values and standard deviation; however, median values and quartile ranges were used for statistical analyses. Differences were considered significant at *p* ≤ 0.05.

## 3. Results

Analysis of Kombucha beverages prepared with black, green, white and red tea at different time points of fermentation revealed the presence of five trace elements (Table 1, Table 2 and Table 3). The content of identified micronutrients manganese (Mn), copper (Cu), iron (Fe), chromium (Cu) and zinc (Zn) was quantified. It should be noted that the composition of separate ingredients such as tea, leaven or sugar was not studied. The general pattern of mineral concentrations in the Kombucha samples was as follows: Zn > Mn > Fe > Cu > Cr.

The content of manganese in Kombucha ranged from 0.43 mg/L to 1.40 mg/L and was dependent on both fermentation time and type of tea used (Table 2). Statistically significant differences are presented in Table 1. The lowest values were observed in Kombucha prepared with black and white tea, while the highest were found in green tea Kombucha. Irrespective of the type of tea used, the highest results were observed on day 14 of fermentation. One liter of the beverage can cover from 19% to 61% of manganese requirement for men and 24% to 78% for women.

The content of copper in Kombucha depended on both fermentation time and the tea used (Table 2) and ranged from 0.01 mg/L to 0.25 mg/L. Kombucha brewed with red tea had the lowest levels of copper, while that prepared with black tea had the highest. Irrespective of the type of tea used, the highest results were observed on day 14 of fermentation, except for white tea (Table 2). Between 7% and 28% of the requirement for this micronutrient was met for both men and women. With respect to this element, there were no statistically significant differences between Kombuchas made with different types of tea on days 1 and 7 of fermentation. The only differences observed were on day 14, leading to the conclusion that the type of tea does not affect copper content in the early days of fermentation.

The content of iron in Kombucha ranged from 0.18 mg/L to 0.46 mg/L and was dependent on both fermentation time and type of tea used (Table 3). The lowest iron levels were observed in Kombucha brewed with green tea, while the highest were found in white tea Kombucha. One liter of the beverage covers as little as 1.8% to 4.6% of the iron requirement for men, and 1% to 2.5% for women.

The chromium content in Kombucha ranged from 0.03 mg/L to 0.09 mg/L and was dependent on both fermentation time and type of tea used (Table 3). The lowest levels were observed in Kombucha brewed with green tea and red (7 days of fermentation). Irrespective of the type of tea used, the highest results were observed on day 14 of fermentation. The requirement for this element was covered at 75% to 232% for both men and women.

The content of zinc in Kombucha ranged from 0.36 mg/L to 2.08 mg/L and was likewise dependent on both fermentation time and type of tea used (Table 4). The lowest levels were noted in Kombucha brewed with white tea, while the highest were found in black tea Kombucha. Irrespective of the type of tea used, the highest results were observed on day 14 of fermentation. The requirement for zinc was covered at 3% to 19% for men and 5% to 26% for women. The mineral content was almost invariably highest on day 14 of fermentation regardless of the type of tea. There were no statistically significant differences between Kombuchas made with different teas on days 1 and 7 of fermentation, so initially the type of tea does not have a significant effect the content of this element.

In addition, the content of micronutrients was analyzed independently of the day of fermentation, taking into account the type of tea used to prepare Kombucha. The highest concentrations of Zn, Cu and Cr were found in the beverage made with black tea. In the case of manganese, the highest concentration of this element was observed in Kombucha brewed with green tea, while that of iron in the Kombucha prepared with red tea (Table 5). The only statistically significant differences were observed for manganese. For the other elements, the type of tea did not affect their content.

Additionally, significant positive correlations were found between some micronutrients (Table 6); however, this relationship was variable for different types of Kombucha. Negative significant correlations were also found for Kombucha prepared on the basis of red tea: Zn vs. Cu and Cu vs. Fe (Table 6).

Regardless of the type of Kombucha, a weak but statistically significant correlation was found between time and the content of Mn (0.279), Zn (0.348) and a moderate, significant relationship between time and the content of Fe (0.423) and Cr (0.447).

Regardless of the day of fermentation, quite strong relationships between the time and concentration of selected mineral compounds were shown in Table 7. Therefore, it can be concluded that both the fermentation process and the time significantly affect the chemical composition of this drink.

## 4. Discussion

Kombucha fermented tea is becoming increasingly popular, not only for its sensory properties, but also for its health-promoting benefits. In addition, the drink is classified as a functional food or nutraceutical [7,30,31].

Our study confirms that Kombucha can be a source of micronutrients: chromium (Cr), manganese (Mn), copper (Cu), zinc (Zn), and iron (Fe). The general pattern of mineral concentrations in the Kombucha samples was as follows: Zn > Mn > Fe > Cu > Cr. Our analysis was carried out at different time points of fermentation (days 1, 7 and 14). In addition, for the first time, different types of leaf tea were used as the base of the beverage: black, green, red and white tea. We have shown that the content of selected micronutrients is dependent not only on the day of fermentation but also on the type of tea used.

Micronutrients are minerals which, although present in trace amounts, are essential for the normal development and functioning of the human body. Given that cells do not have the ability to synthesize trace elements, they must be supplied with food. A properly balanced diet should contain essential minerals in such quantities that the total supply is adequate to meet demand, as both deficiency and excess can cause a range of dysfunctions and disorders [32,33]. The search for and analysis of different types of new foods can add valuable information about a source of micronutrients.

Manganese plays an important role in development, digestion, reproduction, antioxidant defense, energy production, immune response and regulation of neuronal activity [34]. The adult requirement for this mineral ranges from 1.8 mg (women) to 2.3 mg (men) [35]. Our analysis showed that manganese (Mn) concentrations ranged from 0.43 mg/L to 1.40 mg/L, accounting for 19% to 61% of the requirement for this element for men and 24% to 78% for women. The highest manganese content was found in the green tea Kombucha from day 14 of fermentation.

Copper is a component of superoxide dismutase and thus influences free radical decomposition reactions. Moreover, it is responsible for enzymatic reactions and the synthesis of collagen and neurotransmitters [36]. The RDA for adults is 0.9 mg [35]. The amount of copper (Cu) in our study ranged from 0.06 mg/L to 0.25 mg/L on day 14 of fermentation, representing between 7% and 28% of the RDA for both sexes. The highest content of this element was detected in the beverage made with black tea on day 14 of fermentation.

Iron, an essential mineral for health and life, is responsible for the synthesis of hemoglobin—a protein found in erythrocytes, which carry oxygen molecules from the lungs to peripheral tissues and support immune and nerve functions [37]. To prevent iron deficiencies, a daily intake of 10 to 18 mg of this mineral is recommended, depending on life stage and sex [35]. Our Kombuchas contained between 0.18 mg/L and 0.46 mg/L of iron (Fe), representing between 1.8% and 4.6% of the RDA for men and between 1% and 2.5% for women. The highest iron content was found in the beverage prepared with white tea on day 14 of fermentation. The results of the analysis for each beverage revealed differences in the content of minerals, correlated with fermentation time as well as the tea used, and the differences were statistically significant (*p* < 0.05).

Chromium is an essential nutrient for normal metabolism of glucose, protein and fat. It enhances insulin sensitivity in tissues and also participates in intracellular redox reactions. The recommended daily intake of this mineral for men and women aged 19–50 years is 0.04 mg [35,38]. Our results showed that the chromium content in Kombuchas made with infusions from different types of tea ranged from 0.03 mg/L to 0.09 mg/L, meaning that Kombucha can cover as much as 75% to 232% of the RDA for both men and women. The highest content of this mineral was detected in the product made with green and red tea on day 14 of fermentation. Consuming even small amounts of Kombucha will correct deficiencies of this element.

Zinc (Zn) is a key element in many processes, from cell growth and differentiation, to regulating immune system function and modulating mechanisms related to learning and memory [39]. The recommended daily intake is 11 mg for men and 8 mg for women [35]. Zinc was detected in amounts ranging from 0.36 mg/L to 2.08 mg/L on day 14 of black tea fermentation, which accounts for 3% to 19% of the requirement for men and 5% to 26% for women. In addition, it has been demonstrated that *Acetobacter aceti* bacteria biotransform chromium and zinc and increase their amounts. These properties are used in the treatment of diabetes due to their hypoglycemic effect [40].

Minerals such as Mn, Zn and Cu can find be found in plant protection products, fertilizers, pesticides and fungicides. Their presence in the product may be related to agricultural practices in use and the content of these elements in phytosanitary products [41].

Kombucha, despite its long tradition, is not adequately researched. There are few studies analyzing the mineral content of this beverage, particularly in terms of micronutrients. It should be emphasized, however, none of them take into account the different time points of fermentation and type of tea.

Ivanišová et al. assessed the chemical composition and antioxidant, antimicrobial and sensory properties of Kombucha made with black tea on day 7 of fermentation, and their findings appear to be consistent with the present study. Their Kombucha, however, was prepared in a different way. The infusion was made by boiling 1 liter of water, 5 g of black tea leaves (Darjeeling, India) and 30 g of white sugar, left to steep for 15 min and fermented at 22 degrees Celsius for 7 days [30]. In our study, we used 1 liter of water, 100 g of white sugar and 8 g of tea. In the study by Ivanišová et al., the content of manganese was 1.57 mg/L, copper 0.14 mg/L, iron 0.31 mg/L, zinc 0.53 mg/L and chromium was not detected during the analyses. Our results were: 0.67 mg/L for manganese, 0.13 mg/L for copper, 0.24 mg/L for iron, 0.74 mg/L for zinc and 0.04 mg/L for chromium. The most pronounced differences were noted in the content of manganese and the presence of chromium, but this may be related to the use of material of a different origin [30]. The researchers conclude that the content of minerals valuable for the human body increases with fermentation time. In a comparison of the chemical composition and properties of tea and Kombucha, fermented tea proved to be more valuable, and its antioxidant activity was several times higher. During fermentation, the content of the essential elements Fe, Mn, Zn and Ni increased significantly, too. In addition, the authors of the study emphasize that due to the absence of harmful elements, Kombucha is safe to consume [30].

Jayabalan et al. analyzed the chemical composition of the tea fungus (SCOBY) used to brew black tea Kombucha. Their study also made reference to time points, with tests carried out on days 7, 14 and 21 of fermentation. Biochemical properties, including mineral content, increased throughout fermentation time, reaching maximum values on day 21 [2]. Among micronutrients, the highest concentrations in dried tea fungus were found for zinc and manganese. However, the finished Kombucha drink was not analyzed. The results are consistent with our report.

It is worth noting that similar findings were obtained in our earlier study focusing on fluoride ions. In that case, too, longer fermentation resulted in a higher fluoride concentration in the beverage [14].

Similarly, a comparative analysis of sweet black tea vs. Kombucha showed that fermentation significantly increases the content of selected minerals [42]. Concentrations of Zn, Cu, Fe, Mn, Ni and Co were determined. Tests for certain toxic elements showed that Pb and Cr were present in very small amounts, while Cd was not found. Among micronutrients, the highest concentrations were noted for manganese (0.462 ug/mL), iron (0.353 ug/mL), copper (0.237 ug/mL), and the lowest for zinc (0.154 ug/mL) and chromium (0.001 ug/mL). These results are very similar to our observations, with similar concentrations of Mn, Cu and Fe. The Cr content in our study was slightly higher, but the biggest differences were found for Zn. According to Bauer-Petrovska and Petrushevska-Tozi, the levels of copper, iron, manganese, nickel and zinc increase due to the metabolic activity of Kombucha [42].

Tea as such is an important source of elements in the human diet [43]. What is more, the type or species of plant can significantly affect the mineral content in the beverage. This is related, among other things, to the individual differences between species, the diversity of production processes of the plants concerned and the type of soil in which they grow, the capacity of the plants to store nutrients, the types of pollution, climate or geographical location [43,44,45].

Brzezicha-Cirocka et al. analyzed 118 black teas, determining the concentrations of 14 elements. In terms of micronutrients, the highest concentration was found for Mn, along with the highest percentage of the RDA (15%) per daily intake of this beverage [46]. Similar results were also obtained by Koch et al. where, for all black teas tested, mineral contents were ranked in the following order: K > Ca > Mg > Mn > Fe > Na > Zn > Cu. It has been shown that mineral composition can be significantly affected by the origin of black tea, defined not only as a country, but also a region or province [45]. Differences in mineral composition have been noted between black and green teas, and the authors pointed out that apart from production-related factors, mineral content can be also influenced by soil conditions, location, rainfall, altitude, genetic characteristics of the plant, and age of the tea leaves [47].

**Figure 3:**
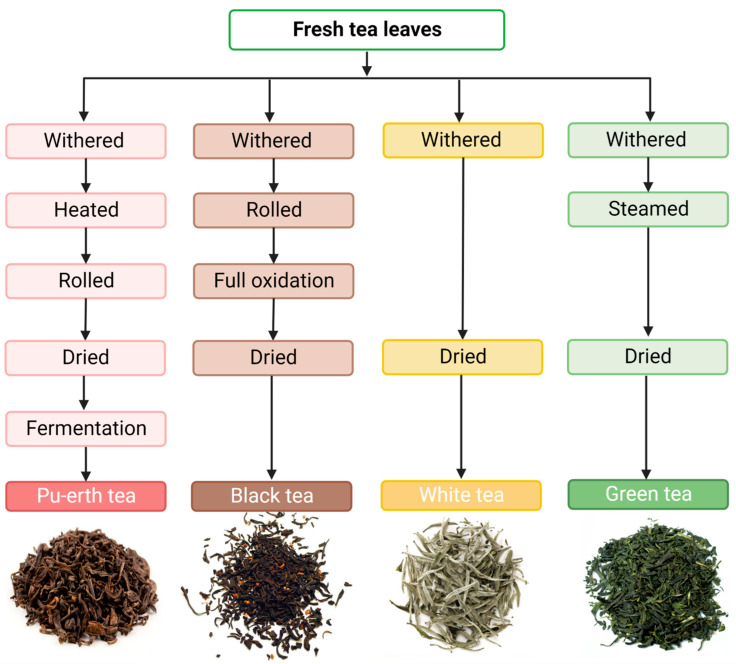
Types of teas and their production process. Created with BioRender.com.

Even though all teas used in this study are derived from the same plant species, *Camellia sinensis*, and come from a single producer in China, they differ considerably in their production processes, as also demonstrated in this paper. Depending on the process followed to obtain the final product, different types of tea can be distinguished. The main types are black tea, white tea, green tea and red tea. Processing treatments affect the color, aroma, taste, intensity, and also the chemical composition of the tea infusion [48]. Black tea is obtained by complete oxidation of tea leaves. After harvesting, the leaves are left to wither completely. During this time, they are also crushed and rolled to speed up the oxidation process. Once they have acquired a sufficiently dark color, they are dried at a high temperature. Red tea is made from green buds and young leaves. Immediately after harvesting, they are heated to inactivate the enzymes. While still moist, the leaves are rolled and then dried in the sun. This type of tea also undergoes fermentation during prolonged storage in high humidity conditions [49]. White tea is obtained from the buds of the tea plant harvested in the spring. Oxidation takes place only where the leaves are damaged, and it is minimal. However, the leaves for white tea are not immediately subjected to a drying process, but are allowed to rest freely, which allows for the activation of enzymatic processes in the leaves, i.e., enzymatic oxidation. Green tea is not oxidized. To make it, withering tea leaves are steamed or pan-fried (Figure 3) [50]. The general principles for obtaining dried tea are very similar, but small differences in production result in distinct flavors and aromas, as well as the chemical composition of the infusion [50].

In our study, irrespective of the day of fermentation, the content of individual micronutrients in Kombucha varied, with the type of tea being the determining factor. The highest concentrations of Zn, Cu and Cr were observed in the beverage prepared according to the traditional recipe, i.e., using the fully oxidized black tea. In the case of manganese, the highest concentration of this element was observed in Kombucha brewed with green tea, while that of iron in the Kombucha prepared with red tea. White tea, which undergoes the least amount of processing, only drying, gentle rolling and light oxidation, has the lowest concentrations of micronutrients. Hence, one of the factors determining the final mineral composition is the processing of the leaves themselves. Tea oxidation appears to be particularly important, as Kombucha made with black tea contained the highest levels of minerals.

It is also worth noting that the micronutrient content increased with the day of fermentation of the beverage, reaching the maximum level on day 14. Today, a significant number of manufacturers embrace the use of fermentation in food production, due to its positive effects on enhancing biosafety, extending shelf life and functional properties [46]. It appears that in most cases, the content of both macro- and micro-nutrients in foods significantly increases during fermentation [51].

The fermentation process increases the bioavailability of micronutrients and trace elements through the degradation of insoluble metal cation complexes and anti-nutritive substances such as oxalates, tannins and phytates [52]. These compounds are hydrolyzed by enzymes (e.g., phytases) produced by microorganisms such as lactic acid bacteria and yeast that make up SCOBY. In addition, the synthesis of lactic acid during fermentation, causing pH changes, provides the conditions necessary for the activation of microbial enzymes, thus contributing to the intensification of their action [53,54]. This mechanism is confirmed by the results of studies by Castro-Alba et al. which showed a correlation between increased availability of iron, zinc and calcium in fermented quinoa flour (3.6, 4.0 and 3.5 times, respectively) and a reduction in phytate levels [55].

Sometimes, microorganisms use individual elements for their own metabolism as a substrate to initiate the fermentation process or the synthesis of secondary metabolites, including vitamins and polyols [56]. We also observed these changes in our study, but they were strongly related to the type of tea and the day of fermentation, which could be related to the activity of microorganisms [51]. Ivanišová et al. reported a decrease in Ca and Pb concentration in the Kombucha drink. Cultures of Kombucha microorganisms show the ability to detoxify the drink, as these bacteria are considered biosorbents. These SCOBY microorganisms have properties that allow them to accumulate and bind heavy metal contaminants on their cellular structure [30]. The results of Mamisahebei et al. showed that the Kombucha cultures used in the beer brewing process are very effective in removing heavy metals such as arsenic, chromium and copper. The content of Co did not increase in Kombucha, probably due to its inclusion in vitamin B 12, as the B vitamins (mainly B 1, B 6 and B 12) are mainly produced during the fermentation process [57].

Scientific studies have confirmed the potential of LAB strains to improve the bioavailability of minerals. The results confirm that the LAB strains LAB *L. fermentum* B4655, *L. plantarum* B4495, *L. casei* B1922, *L. bulgaricus* CFR2028 i *L. acidophilus* B4496 reduced the content of phytic acid when used to ferment soy milk at 37° C for 24 h. In addition, the results show an increase in Mg and Ca levels in fermented soy milk compared to control [58]. Bahaciu et al. [59] showed that germination and fermentation (Lactobacillus) of soybeans for four days at 25 °C led to an increase of 40.87%, 43.41%, 59.56% and 53.4% for Zn, Mg, Fe and Ca, respectively, which are higher than the values obtained for germination alone. Additionally, fermentation (4 or 10 h at 30 °C) of ground quinoa seeds *with L. plantarum* 299v significantly reduced the phytic acid content and improved the bioavailability of minerals such as Ca, Fe and Zn [60]. This direction should be extended in future scientific research.

The limitations of this preliminary study include the lack of composition analysis of the discrete ingredients such as tea, leaven and sugar. These results will provide a complete understanding of the biochemical processes in Kombucha. Nevertheless, it should be noted that Kombucha, especially when subjected to a longer fermentation process, contains more organic acids, and thus its pH is strongly acidic. Moreover, the produced CO2 can start to accumulate between the drinks (Kombucha) and the biofilm (SCOBY). This can prevent the transfer of nutrients and thus block the continuity of chemical changes in the reaction environment. It should therefore be consumed in limited quantities, diluted with water or fermented for a shorter period of time [7].

## 5. Conclusions

Our findings clearly show that the type of tea used to make Kombucha has a significant impact on the micronutrient content of the final product. In addition, fermentation time also determined the levels of selected minerals. Irrespective of the type of tea, the highest results were observed mainly on day 14 of fermentation. Kombucha had the highest content of zinc (0.36 mg/L to 2.08 mg/L), which accounts for between 3% and 26% of the RDA for adults, and the smallest content of chromium (0.03 mg/L to 0.09 mg/L), which, however, represents as much as between 75% and 232% of the RDA. Black tea proved to be the best source of Zn, Cu and Cr; green tea was rich in Mn; while red tea had the highest iron content. In conclusion, Kombucha, particularly based on black tea, can supplement micronutrients in the human diet.

## Figures and Tables

**Table 1:** Analysis of reference material Bovine Muscle NIST-SRM 8414.

Element	Certified [mg/L]	Measured [mg/L] (*n* = 3)	LOD [mg/L]	%RSD Range
Mn	0.37 ± 0.09	0.43	0.00026	1.0–6.2
Zn	142 ± 14	138	0.00065	1.5–5.7
Cu	2.84 ± 0.45	3.06	0.00186	2.4–8.2
Fe	71.2 ± 9.2	76.1	0.00022	1.8–6.7
Cr	0.071 ± 0.038	0.080	0.00044	3.9–9.2

LOD—limits of detection, %RSD range—relative sample deviation.

**Table 2:** The microminerals content (Mn, Cu) and Recommended Dietary Allowances (RDA) in Kombucha.

		Mn (mg/L)					Cu (mg/L)				
Kombucha	Time Points-Day	Mean		SD	% RDA Men	% RDA Women	Mean		SD	% RDA Men	% RDA Women
BK	1 ^a^	0.43 *^,b,c,d,g^	±	0.04	18.87	24.11	0.12 *^,c^	±	0.03	13.00	13.00
	7 ^b^	0.67 *^,a,e^	±	0.13	29.00	37.05	0.13 *^,c^	±	0.07	14.89	14.89
	14 ^c^	0.68 *^,a,f^	±	0.03	29.48	37.66	0.25 *^,a,b,f,i^	±	0.01	28.21	28.21
GK	1 ^d^	1.18 *^,a,e,f,j^	±	0.08	51.25	65.48	0.10 *^,f^	±	0.06	11.26	11.26
	7 ^e^	1.40 *^,b,d,k^	±	0.08	61.03	77.98	0.09 *^,f^	±	0.04	10.17	10.17
	14 ^f^	1.40 *^,c,d,l^	±	0.02	60.88	77.79	0.20 *^,c,d,e,l^	±	0.00	22.20	22.20
RK	1 ^g^	0.93 *^,a,i^	±	0.06	40.35	51.56	0.12	±	0.07	12.89	12.89
	7 ^h^	0.92 *^,i^	±	0.06	39.86	50.93	0.07 *^,i^	±	0.03	7.29	7.29
	14 ^i^	1.21 *^,g,h^	±	0.02	52.66	67.29	0.17 *^,c,h^	±	0.00	18.88	18.88
WK	1 ^j^	0.57 *^,d^	±	0.07	24.97	31.91	0.19 *^,l^	±	0.06	20.90	20.90
	7 ^k^	0.63 *^,e^	±	0.08	27.42	35.04	0.12	±	0.05	13.70	13.70
	14 ^l^	0.71 *^,f^	±	0.00	30.78	30.78	0.064 *^,c,f,j^	±	0.00	7.13	7.13

Different numbers (a–l) in the columns represent statistically significant differences * *p* < 0.05 between particular types of Kombucha (1, 7, 14 days of fermentation) and tea: ^a^—BK 0, ^b^—BK 7, ^c^—BK 14, ^d^—GK 0, ^e^—GK 7, ^f^—GK 14, ^g^—WK 0, ^h^—WK 7, ^i^—WK 14, ^j^—RK 0, ^k^—RK 7, ^l^—RK, BK—Kombucha prepared from black tea, GK—Kombucha prepared from green tea, RK—Kombucha prepared from red tea, WK—Kombucha prepared from white tea.

**Table 3:** The microminerals content (Fe, Cr) and Recommended Dietary Allowances (RDA) in Kombucha.

		Fe (mg/L)					Cr (mg/L)				
Kombucha	Time Points-Day	Mean		SD	% RDA Men	% RDA Women	Mean		SD	% RDA Men	% RDA Women
BK	1 ^a^	0.23 *^,c^	±	0.04	2.27	1.26	0.07	±	0.02	167.19	167.19
	7 ^b^	0.24 *^,c,e^	±	0.03	2.44	1.36	0.04 *^,c^	±	0.01	99.34	99.34
	14 ^c^	0.36 *^,a,b,f^	±	0.01	3.60	2.00	0.07 *^,b,f^	±	0.00	174.09	174.09
GK	1 ^d^	0.25	±	0.09	2.52	1.40	0.04 *^,f^	±	0.02	95.72	95.72
	7 ^e^	0.27 *^,h^	±	0.07	2.68	1.49	0.03 *^,f^	±	0.01	76.28	76.28
	14 ^f^	0.25 *^,c,l^	±	0.01	2.49	1.39	0.09 *^,c,d,e,l^	±	0.00	231.63	231.63
RK	1 ^g^	0.44 *^,h,j^	±	0.24	4.35	2.42	0.05	±	0.01	132.21	132.21
	7 ^h^	0.18 *^,b,e,g,i^	±	0.01	1.81	1.01	0.03 *^,i^	±	0.02	74.53	74.53
	14 ^i^	0.32 *^,h,l^	±	0.00	3.22	1.79	0.09 *^,h,l^	±	0.00	217.51	217.51
WK	1 ^j^	0.19 *^,g,l^	±	0.04	1.90	1.06	0.04	±	0.03	102.34	102.34
	7 ^k^	0.21 *^,l^	±	0.02	2.11	1.17	0.07	±	0.04	180.09	180.09
	14 ^l^	0.46 *^,f,i,j,k^	±	0.00	4.6	2.56	0.06 *^,f,i^	±	0.00	160.32	160.32

Different numbers (a–l) in the columns represent statistically significant differences * *p* < 0.05 between particular types of Kombucha (1, 7, 14 days of fermentation) and tea: ^a^—BK 0, ^b^—BK 7, ^c^—BK 14, ^d^—GK 0, ^e^—GK 7, ^f^—GK 14, ^g^—WK 0, ^h^—WK 7, ^i^—WK 14, ^j^—RK 0, ^k^—RK 7, ^l^—RK, BK—Kombucha prepared from black tea, GK—Kombucha prepared from green tea, RK—Kombucha prepared from red tea, WK—Kombucha prepared from white tea.

**Table 4:** The microminerals content (Zn) and Recommended Dietary Allowances (RDA) in Kombucha.

		Zn (mg/L)				
Kombucha	Time Points-Day	Mean		SD	% RDA Men	% RDA Women
BK	1 ^a^	0.44 *^,a,b^	±	0.30	3.98	5.47
	7 ^b^	0.74 *^,a^	±	0.63	6.73	9.25
	14 ^c^	2.08 *^,a,b,f,i^	±	0.09	18.87	25.95
GK	1 ^d^	0.75	±	0.52	6.80	9.35
	7 ^e^	1.11	±	0.76	10.13	13.92
	14 ^f^	0.54 *^,c,l^	±	0.01	4.91	6.76
RK	1 ^g^	0.88	±	0.65	8.04	11.06
	7 ^h^	0.38	±	0.29	3.44	4.73
	14 ^i^	0.62 *^,c^	±	0.00	5.63	7.74
WK	1 ^j^	0.36 *^,l^	±	0.35	3.26	4.48
	7 ^k^	0.36 *^,l^	±	0.22	3.24	4.46
	14 ^l^	0.99 *^,f,j,k^	±	0.00	8.99	12.36

Different numbers (a–l) in the columns represent statistically significant differences * *p* < 0.05 between particular type of Kombucha (1, 7, 14 days of fermentation) and tea: ^a^—BK 0, ^b^—BK 7, ^c^—BK 14, ^d^—GK 0, ^e^—GK 7, ^f^—GK 14, ^g^—WK 0, ^h^—WK 7, ^i^—WK 14, ^j^—RK 0, ^k^—RK 7, ^l^—RK, BK—Kombucha prepared from black tea, GK—Kombucha prepared from green tea, RK—Kombucha prepared from red tea, WK—Kombucha prepared from white tea.

**Table 5:** The microminerals content in different type of Kombucha.

Kombucha	(mg/L)	Mn	Zn	Cu	Fe	Cr
BK ^a^	mean	0.582 *^,b,c^	1.139	0.185	0.276	0.057
	SD	0.135	0.855	0.088	0.066	0.022
GK ^b^	mean	1.329 *^,a,d^	0.747	0.128	0.253	0.047
	SD	0.129	0.585	0.064	0.065	0.028
RK ^c^	mean	1.034 *^,a,d^	0.647	0.117	0.319	0.053
	SD	0.179	0.428	0.063	0.179	0.026
WK ^d^	mean	0.629 *^,b,c^	0.595	0.127	0.285	0.056
	SD	0.075	0.366	0.068	0.126	0.031

Different letters (a, b, c, d) in the columns represent statistically significant differences * *p* < 0.05 between particular type of Kombucha (1, 7, 14 days of fermentation) and tea: ^a^—BK, ^b^—GK, ^c^—WK, ^d^—RK, BK—Kombucha prepared from black tea, GK—Kombucha prepared from green tea, RK—Kombucha prepared from red tea, WK—Kombucha prepared from white tea.

**Table 6:** Spearman’s rank correlation between micronutrients for different types of Kombucha.

BK:	Mn	Zn	Cu	Fe	Cr	GK	Mn	Zn	Cu	Fe	Cr
Mn	1.00	0.05	0.48 *	0.37	−0.14	Mn	1.00	−0.08	0.37	−0.24	0.18
Zn	0.05	1.00	0.66 *	0.74 *	0.29	Zn	−0.08	1.00	0.00	0.78 *	0.04
Cu	0.48 *	0.66 *	1.00	0.52 *	0.30	Cu	0.37	0.00	1.00	0.16	0.88 *
Fe	0.37	0.74 *	0.52 *	1.00	0.28	Fe	−0.24	0.78 *	0.16	1.00	0.17
Cr	−0.14	0.29	0.30	0.28	1.00	Cr	0.18	0.04	0.88 *	0.17	1.00
RK	Mn	Zn	Cu	Fe	Cr	WK	Mn	Zn	Cu	Fe	Cr
Mn	1.00	−0.19	0.50 *	0.33	0.63 *	Mn	1.00	0.82 *	−0.28	0.76 *	0.37
Zn	−0.19	1.00	0.04	0.51 *	0.25	Zn	0.82 *	1.00	−0.57 *	0.91 *	0.45
Cu	0.50 *	0.04	1.00	0.31	0.45	Cu	−0.28	−0.57 *	1.00	−0.51 *	−0.04
Fe	0.33	0.51	0.31	1.00	0.53 *	Fe	0.76 *	0.91 *	−0.51 *	1.00	0.42
Cr	0.63 *	0.25	0.45	0.53 *	1.00	Cr	0.37	0.45	−0.04	0.42	1.00

* *p* < 0.05, BK—Kombucha prepared from black tea, GK—Kombucha prepared from green tea, RK—Kombucha prepared from red tea, WK—Kombucha prepared from white tea.

**Table 7:** Spearman’s rank correlations between fermentation time and microminerals content for different types of Kombucha.

	BK	GK	RK	WK
Mn	0.712 *	0.712 *	0.659 *	0.554 *
Zn	0.712 *	NS	NS	0.791 *
Cu	0.765 *	0.712 *	NS	−0.844 *
Fe	0.791 *	NS	NS	0.805 *
Cr	NS	0.594 *	0.580 *	NS

* *p* < 0.05, BK—Kombucha prepared from black tea, GK—Kombucha prepared from green tea, RK—Kombucha prepared from red tea, WK—Kombucha prepared from white tea.
